# Exploring the challenges and needs of nursing students in relation to OSCE exam stress: A qualitative study

**DOI:** 10.1371/journal.pone.0327898

**Published:** 2025-07-14

**Authors:** Fahimeh Alsadat Hosseini, Mehrvash Hemati, Maryam Shaygan, Somayeh Gheysari, Azita Jaberi, Mojtaba Ghobadi, Shoeleh Rahimi

**Affiliations:** 1 Community Based Psychiatric Care Research Center, School of Nursing and Midwifery, Shiraz University of Medical Sciences, Shiraz, Iran; 2 School of Nursing and Midwifery, Shiraz University of Medical Sciences, Shiraz, Iran; North-West University Potchefstroom Campus: North-West University, SOUTH AFRICA

## Abstract

**Background:**

The Objective Structured Clinical Examination (OSCE) is a critical assessment method in nursing education but is often associated with significant stress, negatively impacting students’ psychological well-being and academic performance. Understanding the sources of OSCE-related stress and effective coping strategies is crucial for improving nursing education. The aim of this study is to explore the challenges and needs of nursing students regarding OSCE stress.

**Methods:**

Using content analysis, 18 participants (12 nursing students and 6 faculty members) from a nursing school in Shiraz, Iran, were interviewed through in-depth, semi-structured interviews. Data were analyzed using Graneheim and Lundman’s content analysis approach, ensuring rigor through Guba and Lincoln’s trustworthiness criteria.

**Results:**

The analysis identified three main themes: ‘implementing OSCE stress management strategies,’ ‘enhancing OSCE education and assessment processes,’ and ‘preparing students for OSCE.’ These themes reflect the key challenges and effective factors influencing OSCE-related stress among nursing students.

**Conclusion:**

There is a need for comprehensive interventions, including effective stress management strategies, improvements in OSCE training and evaluation methods, and structured preparation programs. Addressing these factors can alleviate OSCE-related stress and enhance student performance. Nursing educators and policymakers should integrate these insights to optimize OSCE implementation, ultimately fostering better psychological resilience and academic success among nursing students.

## Introduction

It is essential for universities to ensure that nursing students possess the requisite knowledge and clinical skills before they are permitted to practice as registered nurses [1,2]. Therefore, evaluating the clinical competencies of undergraduate nursing students is vital prior to their transition into clinical settings, as traditional written assessments are inadequate for measuring clinical proficiency [1]. Alongside ongoing and systematic evaluations of students’ learning throughout their academic program and clinical placements, implementing a practical examination is imperative to verify that students meet the essential professional qualifications [3].

The Objective Structured Clinical Examination (OSCE) was first introduced by Harden in 1975 and has since gained widespread acceptance for evaluating clinical skills within a simulated environment. This contemporary examination format is frequently utilized in health sciences. Rather than being a traditional test, the OSCE serves as an evaluation method encompassing several key components: it comprises multiple stations, each designed to assess different competencies, and all participants are required to navigate through each station. The examination content remains consistent for all students, ensuring that every learner is assessed using identical criteria and predetermined standards [4]. In scenarios involving numerous examiners, supervisors, and stations, students are organized into groups, with each examiner responsible for assessing a specific number of students at their designated station [1].

Currently, the OSCE has been established as an official assessment instrument in the majority of nursing and midwifery programs [5–7]. This evaluation method is characteristically multi-stationed, enabling the simultaneous assessment of various skills and performance levels in a thorough and standardized way. Furthermore, the OSCE facilitates the evaluation of non-cognitive traits, such as perception, stress, confidence, and preparedness, alongside clinical competencies and psychosocial motor skills. This comprehensive approach not only enhances students’ performance and self-assurance but also fosters greater enthusiasm for teaching among clinical educators [8].

Research has demonstrated that the OSCE is a significant source of stress for nursing students [1,9–11]. For instance, Fawaz & Alsalamah (2021:6) noted that although the OSCE may enhance students’ performance and better prepare them for clinical realities, it simultaneously generates considerable stress [9]. A descriptive survey involving 82 first-year nursing students found that the OSCE setting was regarded as stressful, highlighting the necessity for thoughtful support from both academic and clinical faculty [10]. Furthermore, a qualitative investigation by Raziani et al. (2022) involving 25 final-year nursing students revealed that participants faced considerable stress during the OSCE, which potentially hindered their test performance [1].

Test stress represents a biopsychosocial factor that influences both students’ academic performance and overall well-being [12]. Excessive stress can hinder the demonstration of true competence, thereby compromising the validity of the OSCE [13,14]. Elevated levels of test stress may also impede a student’s capacity to derive meaningful learning outcomes from the examination [15]. Moreover, stress associated with testing can result in various adverse effects, including diminished self-esteem, poorer sleep quality, and increased risk of depression and chronic pain [16–19]. Consequently, it is crucial to implement effective stress management techniques and offer psychological support during high-stakes assessments such as the OSCE.

The literature underscores the significance of stress alleviation to enhance students’ learning and practical skills. According to a study by Nematzad et al. (2023) that examined the perspectives of both students and examiners regarding the OSCE, participants emphasized the necessity of employing diverse assessment methods to mitigate the stress experienced by students during the OSCE [20]. Reducing excessive examination stress is beneficial for both educators and students, as it ensures that performance serves as a reliable reflection of actual competence [21] and facilitates optimal learning outcomes. Therefore, exploring OSCE-related stress and anxiety, along with their perceived causes, implications, and meanings for learning, is crucial for developing effective practices in healthcare education.

A variety of studies have investigated different aspects of the OSCE through both quantitative and qualitative approaches in both international and domestic settings. Internationally, quantitative research has largely concentrated on evaluating the effects of OSCE or related programs on student preparedness and performance. For instance, Avraham et al. (2023) assessed the effectiveness of virtual reality programs in enhancing nursing students’ readiness for the OSCE amid the COVID-19 pandemic [22]. In another study, Thabet et al. (2021) explored how the OSCE influences nursing students’ emotional intelligence and their propensity to seek feedback [23]. Additional investigations, including those by Adibone Emebigwine et al. (2023) and Alaskar et al. (2022), have examined the perceptions of health sciences students from various countries regarding the OSCE as a tool for clinical assessment [10,24]. Within the Iranian context, Mojarrab et al. (2020) studied the effects of stress management programs on nursing students’ performance during the OSCE [12]. On the other hand, international qualitative studies have aimed to shed light on the challenges and experiences related to the OSCE. For example, Ataro et al. (2020) performed a qualitative analysis of the views of students and examiners in Ethiopian universities concerning the OSCE [25]. Similarly, qualitative research in Iran has focused on the intricacies and difficulties associated with clinical assessments. Rafiee et al. (2014) conducted a qualitative study investigating the challenges faced by nursing students and faculty in relation to the OSCE [26]. Additionally, Nematzad et al. (2023) examined the experiences of nursing students and faculty regarding the implementation of the OSCE through a qualitative framework [20].

Stress theory, as proposed by Lazarus and Folkman (1984), posits that stress arises from individuals’ appraisal of challenging situations and their perceived availability of coping resources [27]. This theoretical framework is particularly relevant in educational contexts, such as OSCE exams, where students often encounter significant stressors. Applying stress theory offers a valuable perspective for interpreting the findings and gaining deeper insights into the challenges and needs of nursing students related to OSCE exam stress.

Quantitative research has recognized the inherently stressful nature of the OSCE for nursing students [10,28], while qualitative studies have identified stress as a significant obstacle in the OSCE experience [1,20]. However, there is currently a lack of studies specifically investigating the challenges that nursing students encounter concerning OSCE-related stress. This gap underscores the necessity for a more comprehensive understanding of the diverse challenges nursing students face when dealing with OSCE-induced stress. Examining these challenges can yield valuable insights into the particular sources of stress that students experience, thus guiding the development of targeted support strategies and interventions designed to enhance student well-being and performance during clinical assessments. Qualitative research is crucial for revealing nuanced insights and comprehending the complexities associated with various phenomena, significantly enriching the theoretical frameworks that underpin future studies [29]. In this context, investigating nursing students’ experiences of stress during the OSCE is essential for grasping its multifaceted implications. Furthermore, understanding the experiences and viewpoints of both nursing students and their instructors is vital for improving the effectiveness of the OSCE and alleviating the stress students encounter during this assessment process [20]. Therefore, this study aims to explore the challenges and needs of nursing students in relation to OSCE exam stress, thereby contributing to the existing body of literature and offering insights that may inform educational practices and support mechanisms within nursing education.

## Materials and methods

This qualitative study employed conventional content analysis to explore the challenges and needs of nursing students in relation to OSCE exam stress, based on the experiences of undergraduate nursing students who undertook OSCE and faculty members who have administered these assessments at the Nursing and Midwifery School of Shiraz University of Medical Sciences in southwestern Iran. Guided by specific research questions and objectives, this research utilized qualitative content analysis for its systematic and rigorous framework. This approach facilitates the thematic organization of data, allowing for a nuanced understanding of the participants’ experiences and the identification of key themes that emerge from their narratives [30].

This study was conducted between July 4 and December 27, 2023. This qualitative study involved participants from a public nursing school affiliated with Shiraz University of Medical Sciences located in Shiraz, Iran. A total of 18 individuals, consisting of 6 nursing professors and 12 nursing students, were purposefully selected for the study.

Participants were selected using purposive sampling to capture a broad range of experiences related to OSCE-related stress. Inclusion criteria for nursing students were as follows: (1) current enrollment in the Bachelor of Nursing program at Shiraz University of Medical Sciences; (2) being in the third semester of study — an early stage of clinical training — with limited time having passed since their first OSCE experience; and (3) willingness and ability to articulate personal experiences related to OSCE-related stress. Inclusion criteria for nursing faculty members included (1) direct involvement in the planning, implementation, or evaluation of OSCEs; (2) a minimum of two academic semesters of experience working with students in OSCE contexts; and (3) willingness to share their insights regarding students’ stress during OSCEs. Exclusion criteria for both groups included unwillingness to participate or inability to clearly express their experiences due to emotional, psychological, or medical conditions (e.g., recent trauma or acute distress), as subjectively assessed by the research team during initial contact.

Upon receiving ethical approval for the study, the lead researcher approached individuals who fulfilled the inclusion criteria and expressed their willingness to participate. Prior to each interview, participants were briefed on the study’s objectives, the rationale behind audio recording the sessions, the confidentiality measures regarding their data, and their right to withdraw from the research at any point. They were also informed that their involvement was entirely voluntary. Written informed consent was secured from each participant, indicating both their voluntary participation and their authorization to record the interviews.

Although the term “exam stress” has been widely addressed in previous literature, this study did not adopt a pre-defined operational definition. Instead, in line with the principles of qualitative content analysis, stress was conceptualized as an emergent construct derived from participants’ subjective accounts and real-life experiences during the preparation for and performance of the OSCE.

Data were gathered through individual face-to-face semi-structured interviews lasting between 30 and 45 minutes, conducted in a quiet environment within the nursing school. The potential for participants to provide rich, descriptive accounts was assessed subjectively, focusing on the depth and clarity of their responses to open-ended questions during the interviews. The data collection continued until thematic saturation was reached. All interviews were conducted by the first author, a nursing faculty member with formal training in qualitative research and prior experience in conducting interviews with nursing students. Each interview commenced with semi-structured and open-ended questions, such as, “Can you describe any stressful challenges you have faced as a nursing student during the OSCE?” “Which components of the OSCE do you find most stress-inducing, and what do you believe contributes to this stress?” and “What forms of support do nursing students require to alleviate their stress related to the OSCE?” Follow-up questions were tailored based on the participants’ answers to gain a deeper understanding of their experiences.

Follow-up questions were formulated in response to the participant’s feedback to obtain a deeper understanding of the context. Probing inquiries, such as “Could you elaborate on that?” and “Can you provide an example?” were employed to encourage further elaboration. Additionally, clarifying questions, including “What do you mean by that?” were posed as needed to ensure clarity in the participant’s responses. The interview guide was specifically developed for this study, and an English version is provided as a supplementary file (S1 File).

To ensure the face validity of the semi-structured interview guide, it was initially reviewed by two faculty members with expertise in qualitative research and nursing education. Subsequently, the research team held internal discussions to evaluate the clarity, logical flow, and alignment of the questions with the study objectives. Minor refinements were made based on expert and team feedback. Furthermore, a pilot interview was conducted with two nursing students from the target population to assess the comprehensibility, relevance, and feasibility of the interview questions. Based on their feedback, additional adjustments were made to enhance clarity and flow.

The data gathered were subjected to analysis using the content analysis framework established by Graneheim and Lundman. This analysis was conducted in an inductive manner, with themes and categories emerging from the data rather than being predetermined. The process began with open coding, where the transcripts were read repeatedly to identify meaningful units of text. These units were then assigned initial codes that captured their essence.

Subsequently, codes were grouped into subthemes based on their similarity, which were further refined into broader categories. Finally, these categories were condensed and abstracted into themes that best represented the key challenges and needs of nursing students experiencing OSCE exam stress.

Initially, the researchers transcribed the interviews verbatim and reviewed the texts multiple times to gain an in-depth understanding of the material. Each interview was considered a unit of analysis and was meticulously examined for significant meaning. The transcripts were then broken down into meaning units, which included individual words, sentences, or paragraphs that represented relevant information in relation to the research questions.

The meaning units were assigned initial codes that captured the essence of the text. Subsequently, these codes were compared and contrasted for similarities and differences, leading to their organization into subthemes. These subthemes were further abstracted into categories, and through a process of constant comparison and reflection, the final themes were developed.

The process was inductive, meaning that the analysis was guided by the data itself rather than pre-existing theoretical concepts. This allowed for the identification of emergent themes grounded in the participants’ experiences [31].

To uphold the validity and reliability of the interpretation and analysis of findings, a series of rigorous procedures were implemented throughout the research. The criteria for trustworthiness established by Guba and Lincoln [32], which encompass credibility, transferability, dependability, and confirmability, were systematically adhered to. Specifically, member checking was conducted to review and validate initial findings with a select group of participants, ensuring that the results accurately reflected their experiences. Moreover, the coding and thematic analysis were carried out by two independent researchers to improve inter-rater reliability and reduce potential biases, with any discrepancies addressed through consensus. The research team also participated in peer debriefing sessions to obtain external insights into the data interpretation. Thick descriptions and comprehensive documentation of the analysis process were maintained to support transferability and facilitate replication of the study. Additionally, strategies such as prolonged engagement, negative case analysis, and diverse sampling were employed to strengthen the trustworthiness of the data. A collaborative approach was adopted to ensure that the categories genuinely represented participants’ perspectives, involving ongoing discussions and consensus-building throughout the analytical process.

### Ethical considerations

Upon obtaining approval from the Ethics and Research Committee at Virtual University of Medical Sciences (Approval No. IR.VUMS.REC.1401.034), participants who fulfilled the study criteria were selected. Both verbal and written informed consent were obtained from all participants after providing oral and written explanations of the research objectives. To address any potential emotional discomfort related to sharing personal experiences, participants were assured of their right to withdraw at any point during the study and were provided with information about counseling services if required. Additionally, confidentiality was meticulously maintained through the anonymization of data, safeguarding the identities of participants throughout the research process

## Results

A total of 18 participants were involved in the interviews, which included 12 (66.67%) nursing students and 6 (33.33%) faculty members from the Nursing and Midwifery School at Shiraz University of Medical Sciences. All student participants were in the third semester of the bachelor’s program—corresponding to the second academic year—and had recently completed their first OSCE. This shared academic stage provided a consistent background for exploring their experiences of OSCE-related stress. The nursing faculty members had expertise in various fields, including mental health nursing (n = 1), pediatric nursing (n = 2), community health nursing (n = 1), and medical-surgical nursing (n = 2). The majority of the participants were female (72.22%) and single (66.67%). The mean age of the faculty members was 44.67 years (SD = 8.29), while the mean age of the nursing students was 20.67 years (SD = 0.89). The mean work experience of the faculty members was 13.67 years (SD = 6.53). Table 1 presents the characteristics of the study participants.

**Table 1:** Characteristics of the study participants.

Participant	Gender	Age (years)	Marital status	Professional position
P1	Female	54	Married	Nursing faculty
P2	Female	37	Single	Nursing faculty
P3	Female	38	Married	Nursing faculty
P4	Female	56	Married	Nursing faculty
P5	Female	40	Married	Nursing faculty
P6	Female	43	Single	Nursing faculty
P7	Male	23	Single	Nursing student
P8	Male	21	Single	Nursing student
P9	Male	21	Single	Nursing student
P10	Male	21	Single	Nursing student
P11	Female	20	Married	Nursing student
P12	Female	20	Single	Nursing student
P13	Female	20	Married	Nursing student
P14	Male	21	Single	Nursing student
P15	Female	20	Single	Nursing student
P16	Female	21	Single	Nursing student
P17	Female	20	Single	Nursing student
P18	Female	20	Single	Nursing student

The results were categorized into three main themes: “implementing OSCE stress management approaches”, “improving OSCE education and evaluation”, and “preparing students for the OSCE”, along with 11 associated categories. An overview of these main themes and categories is presented in Table 2.

**Table 2:** Main themes, categories, and subcategories addressing students’ challenges and needs related to OSCE stress.

Main theme (Need)	Category (Need)	Subcategory (Challenge)
Implementing OSCE stress management approaches	Reducing OSCE high stress	Significant inevitable stress
		High stress in the first experience and exposure
		Ongoing OSCE high stress
	Providing a needs-based program	Preliminary assessment of OSCE stress management needs
		Targeting the needs for OSCE stress management in students
	Developing time management skills	Perceived OSCE time pressure
		Mismanagement of OSCE time
	Addressing negative attitudes	Negative attitudes and misconceptions
		Lack of self-efficacy and confidence
	Creating a peaceful atmosphere	Lack of a peaceful, supportive environment before the exam
		Need for instructors’ peaceful presence during the OSCE
Improving OSCE education and evaluation	Improving the OSCE educational process	Educational shortcomings
		Excessive instructional materials
		Inconsistent exam-related content delivery
	Introducing and advancing OSCE evaluation procedures	Limited familiarity with the OSCE evaluation process
		Stress-inducing grading system
	Upgrading educational and technological infrastructure	Inadequate educational infrastructure
		Inadequate technological infrastructure
Preparing students for the OSCE	Awareness of the exam process	Unfamiliar and unpredictable nature of the OSCE
		Uncertainty about the OSCE-related environment and expectations
	Previous exam experience	Lack of pre-OSCE experience
		Request for experiencing a pre-OSCE simulated environment
	Pre-OSCE practice and preparation	Limitations in frequent use of pre-OSCE practice space
		Need for pre-OSCE practice with diverse, effective methods and feedback

### Implementing OSCE stress management approaches

The theme “Implementing OSCE stress management approaches” includes five categories: “Experiencing and addressing high OSCE stress”, “Developing time management skills”, “Providing a needs-based program”, “Addressing negative attitudes,” and “Creating a peaceful atmosphere”. Participants identified effective stress management as a critical need for nursing students during the OSCE, highlighting their frequent high stress levels. They emphasized the importance of a tailored stress management program to address this issue. Additionally, students encountered time management challenges and held negative attitudes towards the examination, themselves, and their future prospects. Addressing these factors, along with creating a peaceful atmosphere, is essential for mitigating OSCE-related stress.

#### i. Reducing OSCE high stress.

Based on the participants’ interviews, a clear need to reduce high OSCE-related stress among students was revealed. Nursing students and faculty agreed that experiencing stress during the OSCE is unavoidable, particularly at the beginning of the exam, especially for those who are encountering it for the first time. Participants noted that students at both extremes of academic performance—those excelling and those struggling—experience heightened levels of stress. As one faculty member stated:

*“When they first enter, everyone is really confused and anxious... but this stress is actually unavoidable; they all feel it at the beginning” (P.4).*

The OSCE is associated with elevated stress levels, particularly for first-time examinees. One student commented, *“One of the drawbacks of the OSCE test is that students’ stress is very high during the exam, particularly for those taking it for the first time” (P.6).*

Additionally, some students reported particularly high stress levels, especially those who perform well academically. One participant shared:

*“Some of them have a very high level of stress, particularly those who do really well in their studies... Those who enter the stations as the first group also have elevated stress levels, while those in the last group tend to be more relaxed. However, overall, their stress is quite high, whether because they have studied a lot, are diligent students for whom this score is very important, or due to a lack of preparation” (P.3).*

Faculty members believed that high levels of stress could negatively impact not only students’ mental well-being but also their examination results.

It has been noted that stress levels can become so overwhelming that students often feel disoriented, with some even crying before or during the exam. This intense stress frequently undermines their test scores.

*“Some students experience such high levels of stress and pressure that it leads to forgetfulness regarding their processes and training” (P.5).*

There are students whose failure to pass the exam can be attributed to stress and anxiety.

*“During the six minutes they are expected to respond, they become completely immobilized and are unable to articulate anything, even those who are usually high-performing students” (P.2).*

Student interviewees also acknowledged the negative effects of stress on their performance during the exam. One student articulated this by stating, *“Stress has a significant impact on the process we want to execute because it is a practical task, and stress can affect us to the point where our hands shake and everything goes awry”* (P.9)

One of the most significant challenges faced by nursing students during the OSCE was that poor performance at one station led to ongoing and compounded stress in subsequent stations, negatively affecting their performance. In this regard, one nursing student remarked:

*“Imagine I run out of time at the first station; my mind remains focused on that first station, and I start feeling stressed. I can no longer perform at the other stations; I think, ‘Oh, I messed that one up; I’ll ruin the rest in order” (P.11).*

In this context, one nursing faculty member emphasized not only the detrimental and ongoing impact of stress on students’ performance during the exam but also the necessity for students to learn stress management skills. The faculty member stated:

*“Most students provide feedback that when, especially, our first stations go poorly, it affects the subsequent stations that we knew how to handle as well. I believe students need to learn this skill: to not carry their stress with them until the end of the exam” (P.6).*

Another nursing faculty member highlighted the importance of implementing pre-examination measures to enhance stress management for the OSCE among students. The faculty member noted:

*“If we could do something for the students beforehand to help them manage their stress before entering this situation, it would certainly be better. They might be overwhelmed or unsure about how to manage their stress. If a week prior, this individual has a foundational personality, they should continuously engage in certain activities. The simplest recommendations we can provide include relaxation techniques, deep breathing, maintaining focus, and meditation” (P.1).*

#### ii. Providing a needs-based program.

Participants emphasized that the OSCE stress management program should be tailored to meet the specific needs of students. They indicated that students in need of such programs must be identified in a targeted manner.

One participant suggested, *“These anxious students should be identified early on by their academic advisors. From the first semester, university psychologists can also help identify them through conversations. Eventually, workshops on stress management and confidence building should be organized for them. They should be provided with educational resources, including relevant books for self-study and practice to help reduce their stress” (P.10).*

Another participant noted, *“It is essential to identify anxious students based on specific questionnaires. Subsequently, interventions and preparations can be tailored to their personality traits” (P.2).*

Moreover, participants stated that the content design should be based on a comprehensive needs assessment conducted among students, with the delivery of this content taking place before the examination when students are most in need of such programs. As one participant articulated, *“It would be beneficial to conduct a needs assessment since the OSCE evaluates students’ knowledge, attitudes, or skills. An initial needs assessment should be performed... Working on these aspects before their exam can be very helpful” (P.2).*

#### iii. Developing time management skills.

Participants identified time pressure as one of the key challenges contributing to examination stress. One student expressed, *“The time allotted for the OSCE is truly frustrating; it feels like you can hear the ticking of the seconds, and it’s incredibly stressful” (P.12).*

Nursing faculty members also noted that the time constraints for completing required procedures during the OSCE stations significantly heighten students’ stress. They emphasized the necessity for students to develop effective time management skills. As one faculty member stated, *“One of the stress factors in the OSCE is time management... Students may know the procedures well, but when it comes to managing their time, such as completing a procedure in three or five minutes, they become so flustered that they can’t perform. They are solely focused on the time pressure and worry that they won’t finish in time. One possible solution could be to have them practice their procedures with a time management focus from the outset, ensuring they adhere to the time limits rather than the durations they practiced previously” (P.6).*

#### iv. Addressing negative attitudes.

From the participants’ perspective, one of the significant challenges faced by nursing students was the presence of negative attitudes and misconceptions. Interviewees indicated that receiving stress-inducing and sometimes inaccurate information from students with prior OSCE experiences creates a negative outlook and preconceptions about the exam, leading to increased stress.

One faculty member remarked

*“Previous candidates who have taken the OSCE instill stress in current students by saying, ‘Remember, in section X and field Y, especially if it’s certain instructors, keep this in mind.’ This leads to preconceptions among these students, formed by the experiences of others, exacerbating their stress about the exam. It would be beneficial to hold sessions where students can voice their challenges in a Q&A format, providing them with answers to correct those inadequate or negative attitudes” (P.5).*

Similarly, one student referred to the stress-inducing information received from upperclassmen regarding the OSCE, suggesting that obtaining information directly from the relevant instructors would help alleviate such stress:

*“The upperclassmen scared us a lot before the midterms. They would say things like, ‘If you fail, this is what will happen,’ which really frightened us. I think students shouldn’t ask upperclassmen at all. Instead, if they ask the instructors, it would be much easier for them to manage their stress than relying on upperclassmen” (P.13).*

One of the most critical challenges and concerns for nursing students, as identified by interviewees, was the stress stemming from the fear of failing the exam and its subsequent consequences. As one participant articulated, *“Some individuals’ lives depend on this exam, determining whether they will graduate or not. They worry about how their peers will perceive them; if a student fails the exam, they fear others will think they haven’t learned anything over these four years or that they lack the competence to graduate, which significantly impacts their self-esteem” (P.5).*

Participants underscored the importance of fostering self-efficacy and confidence in students when facing the OSCE. One interviewee emphasized. One interviewee emphasized,

*“Another important aspect is the lack of trust that students have in themselves and their abilities. If a student knows they have studied well, have a good memory, have put in the effort, and have gained sufficient knowledge during their internships, they will naturally experience less stress” (P.1).*

#### v. Creating a peaceful atmosphere.

Participants believed that nursing students require a supportive and calming environment to effectively cope with the stress associated with OSCEs. The interviewees asserted that establishing a tranquil atmosphere and implementing various stress-reducing measures prior to the examination in the quarantine room (waiting room) could significantly alleviate exam-related stress among students.

One participant commented, *“The psychological state within the quarantine is very important. If there is someone to calm them down, this is crucial and helps lower their stress levels” (P.3).* Another stated, *“The quarantine space is not suitable. A more open environment should be considered. After all, students have to stay in a confined space for several hours. If possible, they could have a varied program—watch a movie, something entertaining to distract them and reduce their stress” (P.4).*

A third participant expressed, *“Many of them genuinely experience high levels of stress. Especially for those who are in the last group, the quarantine period extends longer. A solution must be found to help them relax during this time, whether through music or relaxation training techniques to manage their stress” (P.6).*

Moreover, they noted personal strategies for managing stress: *“Everyone has their way of reducing stress; some read books, others listen to music. Personally, I never study an hour before my exam; I just listen to music or read a comforting verse, which helps calm my heart. Hearing the sound of water and seeing natural scenery also alleviates my stress. I perform ablutions, which helps reduce my stress. The sound of the call to prayer, prayers, and certain verses provide me with peace... Before the OSCE, we recite a prayer, and then we send blessings. This helped us feel very calm” (P.11).*

Additionally, the calming presence of instructors played a significant role in reducing stress and enhancing student performance during the OSCE. Interviewees believed that having familiar instructors at the stations, along with their calming demeanor, was crucial for alleviating student stress.

*“I always prayed to be with my own instructor. I had done these exercises with my own instructor in class; I know what to expect. If I were with another instructor, I would not know their methods, which would add to my stress” (P.14).*

*“We usually assign clinical instructors for the OSCE who are familiar to the students, and this familiarity greatly helps them manage their stress. However, we have occasionally had instructors who were not familiar to the students, and they provided feedback that they felt more anxious when with those instructors” (P.6).*

*“It seems that knowing there is a familiar instructor helps reduce their stress a bit... Instructors should be cheerful at the stations. Yes, there is fatigue, but from start to finish, they should maintain a cheerful demeanor because the student is not responsible for our fatigue. They prefer to see their instructor’s vitality rather than fatigue or impatience. This can make the students feel nervous and lose their composure, so it’s essential to provide them with calmness... Especially for the first group, who need more reassurance. For example, instructors should greet them and engage in small talk, and this can significantly reduce their stress” (P.3).*

*“All of them experience this stress initially. I believe the instructor can provide significant comfort... When the students first enter, they are quite disoriented and anxious. I tell them to stay calm; this is the station, this is the scenario, and we help them with a brief introduction” (P.4).*

Furthermore, students preferred that the demeanor of the supervising instructors during the OSCE be uniform and characterized by a calm and neutral approach. *“The way instructors interact significantly influences the exam experience. I think all instructors should be consistent in their approach, maintaining a neutral and calm demeanor—neither overly encouraging nor excessively harsh, which could exacerbate stress” (P.7).*

### Improving OSCE education and evaluation

Another key theme identified in this study regarding the challenges and factors influencing stress related to the OSCE in nursing students was “improving OSCE education and evaluation”. This main theme encompasses the sub-themes of “improving the OSCE educational process”, “introducing and advancing OSCE evaluation procedures”, and “upgrading educational and technological infrastructure”. The interviewees emphasized the need for enhancements in the educational and evaluative processes as well as in the underlying educational and technological infrastructure for this examination.

#### i. Improving the OSCE educational process.

Participants in this study expressed a need to enhance the educational processes associated with the OSCE to reduce students’ examination-related stress. Participants mentioned several educational challenges, including educational shortcomings, excessive instructional materials, and inconsistent exam-related content delivery. Both faculty members and nursing students believed that addressing these challenges could be effective in reducing OSCE stress.

In discussing the need for improving educational quality, two nursing faculty members stated:

*“When education is hybrid, it works better. For instance, when you are supposed to learn a particular subject, no matter how much you read, the instructor can quickly clarify things in just a few words, making it easier to grasp. However, lectures have a drawback as they lack repetition, while in online formats, students can review the material multiple times and gain reassurance. This method reduces students’ stress on the exam day” (P.4).*

*“Some of the students’ stress stems from the quality of education provided by the institution, which must be adequate” (P.5).*

One student commented on the necessity of reforming the OSCE educational process and the stress caused by the extensive volume of materials:

*“Another limitation we faced was the difference between what is taught to thirty students in class and the experience of performing in front of an instructor who evaluates your work directly. I suggest that OSCE training be conducted separately for each group. There are significant differences between what is real and what occurs in the classroom or practice hall. For instance, setting up a scenario is one of the objectives of the OSCE, and we often lack many tools, or the equipment provided is not up to standard, making it difficult to understand the flow of the process. We weren’t even familiar with the names of many tools, which presented several limitations. I believe that before students undertake the OSCE, they should be taken to a real environment where they can see how the five procedures they learned are actually performed to gain a comprehensive view... The volume of materials is overwhelming and stressful” (P.12).*

Interviewed students expressed that uniformity in the academic levels of instructors during training could significantly mitigate their stress levels during the OSCE:

*“The teaching levels of instructors should be similar so that there is little variation among students, preventing discrepancies that could induce stress during examinations” (P.9).*

#### ii. Introducing and advancing OSCE evaluation procedures.

In this study, interviewees indicated that limited familiarity with the OSCE evaluation process and grading system contributed to their OSCE-related stress. They believed that introducing clearer OSCE evaluation processes and reforming the grading system would significantly reduce learners’ stress.

Nursing faculty expressed the following views:

*“Perhaps there should be some explanation about the OSCE, such as how the setup works and how the exam is conducted. Familiarity with the OSCE, its stages, and grading procedures, or information regarding these aspects, could help alleviate students’ stress to some extent” (P.6).*

*“It would also be beneficial if the OSCE were not an entirely unfamiliar process. Discussing it with the students beforehand could help eliminate fear and stress. Additionally, if the scoring were limited and divided into two or three separate assessments, students would not experience as much stress” (P.4).*

*“It may be necessary to allocate a larger portion of the OSCE score to the practical internship grades. This would essentially lead to continuous evaluation, preventing students from facing overwhelming stress all at once when their performance could determine their future... We should observe how assessments are conducted in advanced countries and draw lessons from them” (P.5).*

Two of the interviewed students emphasized the importance of familiarity with the evaluation process and its improvement:

*“There should be explanations provided about the exam, not just on the day of the test. Gradually informing students from the first session about the conditions and grading criteria would be helpful. For instance, we used to go in without knowing what to expect or on what basis we would be graded, and very little had been discussed about it, which caused us stress” (P.10).*

*“Our checklist differs from the one used in the exam. There are several important items that are not included in our checklist, and not knowing the evaluation criteria is stressful. There shouldn’t be any discrepancies” (P.7).*

#### iii. Upgrading educational and technological infrastructure.

Participants referred to the inadequate educational and technological infrastructure of the OSCE. They highlighted the necessity of developing and enhancing educational and technological infrastructure as a critical factor in managing OSCE exam stress.

Students involved in the interviews reported that challenges related to educational facilities, such as inadequate classroom space, insufficient or faulty equipment, and a lack of familiarity with using these tools, significantly hindered their ability to engage in adequate practice for the OSCE, leading to increased stress during exams. One student noted:

*“Most of the stress comes from insufficient practice. We cannot engage in proper training due to time constraints or overcrowded classes. Often, we want to practice, but the classes are full, and the equipment is either broken or difficult to use” (P.15).*

Additionally, the disparity between the equipment provided during practice sessions and that encountered in actual exam scenarios was identified as a significant source of stress. One student recounted:

*“When we were preparing for the OSCE, we were given a mannequin that none of us knew how to operate. I would say 95% of us were unable to perform because we had no idea how to work with the mannequin, and this was a source of stress for us” (P.12).*

Participants expressed the need for improved educational support materials to enhance the skills necessary for nursing students during the OSCE and, consequently, reduce their exam stress. One participant articulated:

*“One limitation we face is the lack of educational aids. For instance, instructors could demonstrate specific procedures, record them, and make these videos available online for students to reference. After the instructor demonstrates a procedure, the students often look for someone else to teach them, but there is no one available to provide that instruction as effectively as the instructor did. This gap in resources means we forget many details. Having access to instructional videos for all procedures would be beneficial, as it would alleviate our stress about performing them correctly” (P.8).*

Furthermore, participants believed that incorporating technology into OSCE education was vital, yet they underscored the necessity of establishing the requisite infrastructure to adequately prepare learners. One nursing faculty member remarked:

*“I believe students are eager to utilize new technologies; however, we lack the necessary facilities. Anything related to digital technology and the internet inherently involves challenges such as budget constraints, insufficient resources, the need for specialists to design and support these technologies, as well as issues with internet connectivity and speed. These factors need to be addressed. Additionally, the content must be engaging enough for students. We must provide adequate orientation to ensure that students can use the technology without stress, and the interface must be user-friendly to minimize their concerns about engaging with the technology. When we provide such resources, student performance is likely to improve, and their stress will decrease” (P.1).*

### Preparing students for the OSCE

Participants in this study identified “preparing students for the OSCE” as one of the most significant themes and challenges contributing to OSCE exam stress. This main theme encompasses three sub-themes: “awareness of the exam process,” “previous exam experience,” and “pre-OSCE practice and preparation”. Participants expressed that students needed to acquire knowledge about the exam process, experience a simulation similar to the OSCE, and engage in practice and preparation for the exam procedures.

#### i. Awareness of the exam process.

Participants believed that one of the main challenges for nursing students was a lack of sufficient awareness regarding the OSCE examination process. In this regard, nursing faculty remarked:

*“A part of their stress stems from how the exam is conducted” (P.6).*

They further suggested that students should not face an unknown process during the OSCE and emphasized the importance of discussing the exam in advance to alleviate fear and stress. One instructor noted:

*“They often feel anxious, especially if the OSCE is critical. It would be beneficial to familiarize them with the exam beforehand, either virtually or in group settings. We should explain how the OSCE works and provide them with examples of OSCE exams so that they understand its format. Under these circumstances, their stress would likely be reduced” (P.4).*

Nursing students also highlighted the importance of understanding how the OSCE is conducted. One student shared:

*“First of all, we have never seen how the exam is administered, and we have no background in it. Personally, I felt stressed because I didn’t know how we would be evaluated... I was completely unfamiliar with the concept of quarantine. We didn’t know we weren’t supposed to have our phones, and suddenly there was a quarantine; it was a shock for everyone... I suggested to the instructors that they provide us with a video or content so that we truly understand what we need to do” (P.11).*

Two students expressed a desire for students to be shown the exam environment before the OSCE, stating:

*“Not knowing what to expect is very frightening” (P.9).*

*“The mere mention of quarantine is intimidating; people don’t know what it entails or what will happen. That uncertainty about the process contributes to our stress” (P.18).*

#### ii. Previous exam experience.

One of the stressful challenges identified by participants regarding the OSCE exam was the lack of prior experience with pre-OSCE simulations among nursing students. Participants believed that experiencing a situation similar to the OSCE significantly contributes to reducing their exam stress. They acknowledged the advantages of utilizing simulated exam environments, whether in-person or virtual, to create prior exam experiences for nursing students. One participant emphasized:

*“It is unfortunate that we haven’t had the opportunity to experience an OSCE-like situation before the main OSCE. Unless students find themselves in that situation, they cannot manage this stress. It would be beneficial to provide an environment that closely resembles the real setting or to employ simulation methods that allow them to envision themselves in those circumstances; this could help them manage their stress to some extent” (P.6).*

Another participant mentioned:

*“If the simulation process is conducted experimentally before the OSCE and students experience the situation even once, their stress levels will definitely be much lower” (P.3).*

Another student stated that:

*“Simulating the conditions is much better than suddenly being placed in a situation with no information; this way, stress increases significantly” (P.16).*

#### iii. Pre-OSCE practice and preparation.

Participants in this study identified the challenge of limited access to the Pre-OSCE practice space. Moreover, they highlighted new technologies as a useful method for overcoming the limitations of in-person practice. One participant noted:

*“Practicing a lot indeed reduces stress; however, the practical classes might not always be available, or it might not be feasible to attend often. But if we can utilize virtual reality, we could perform the exercises more frequently using virtual reality headsets. This would undoubtedly enhance our skills and lessen our stress” (P.8).*

The nursing students involved in this study expressed that employing diverse practice methods before the exam and obtaining feedback from experienced individuals regarding the execution of procedures could enhance their learning and alleviate stress. One student elaborated on this:

*“Practicing is very important for my stress reduction. However, I suggest that the practice should not be repetitive. You know, it shouldn’t just be that a model is provided and you are told to perform the same task ten times. For example, they could say, ‘Do this procedure on your neighbor’s arm once, then let your neighbor perform it on you.’ Or one time in front of the instructor, and another time collectively... It would be beneficial to start from the beginning and have an upper-class student act as a mentor, pointing out your mistakes and teaching you what to do, or even gathering the group half an hour before the exam to explain the procedures to each other. When several people explain a task, it reinforces the information, makes it more memorable, and reduces stress” (P.17).*

## Discussion

Nurse students widely recognize the OSCE as a significant source of stress [1,9–11]. Such stress not only undermines academic performance but also adversely affects students’ mental health and overall well-being [12]. This study addresses the challenges and needs of nursing students in relation to OSCE exam stress, contributing to the body of literature and offering insights to provide supportive strategies for nursing students.

In the current study, the main challenges and then needs of nursing students related to OSCE exam stress were explored. The main needs of nursing students in relation to OSCE exam stress in this study were organized into three main themes: “Implementing OSCE stress management approaches”, “Improving OSCE education and evaluation”, and “Preparing students for the OSCE.”

### Implementing OSCE stress management approaches

The findings indicate that managing OSCE stress is a significant challenge for nursing students, with this theme comprising five categories: “Reducing OSCE high stress”, “Providing a needs-based program”, “Developing time management skills”, “Addressing negative attitudes”, and “Creating a peaceful atmosphere.”

Participants have an essential need to reduce high OSCE-related stress. Participants frequently reported significant stress during the OSCE, which negatively impacted their performance. This finding aligns with the literature, where the OSCE is consistently associated with high levels of stress [33,34]. For instance, research by Simbolon and Simbolon (2024) found that over 70% of students experienced severe stress during the OSCE [35]. Similarly, Majumder et al. (2019) reported that a significant portion of students (67–79%) found the exam intimidating [36]{Majumder, 2019 #3283;Majumder, 2019 #3283}. Nevertheless, some studies, such as that by Martin and Naziruddin (2020), have argued that OSCE-related stress does not significantly influence performance outcomes [37]. However, differences in participants, settings, and the educational and evaluation processes related to the OSCE in this study compared to other studies may have contributed to these results. The nature of the OSCE itself contributes to heightened stress, as it involves being under direct observation and assessment with no opportunity for corrective actions. The test’s one-time, irreversible nature exacerbates stress levels, making it more stress-inducing compared to other assessment formats [12]. High levels of stress were found to disrupt student performance. This finding is consistent with previous research, which has shown that elevated stress levels before the test can impair focus, cause confusion, and reduce academic performance [1,9,12,38]. Stress has been noted to hinder cognitive processing and negatively affect essential performance parameters, such as knowledge application, attitude, and psychomotor skills [39]. These insights underscore the need for effective stress management strategies tailored specifically to the unique stressors associated with the OSCE.

The findings of the current study highlight a critical need for a tailored stress management program specifically designed for nursing students. Numerous studies have underscored the necessity of providing adequate support to assist nursing students in coping with stress related to the OSCE [10,12,40–43]. Aligned with the results of this investigation, previous research has explored various interventions aimed at mitigating test stress among nursing students. These interventions include techniques such as progressive muscle relaxation, guided reflection, cognitive restructuring, thought-stopping strategies, the use of earplugs, and aromatherapy [40–43]. Mojarrab et al. (2020) reported that their stress coping program effectively reduced stress levels and subsequently improved students’ performance in the OSCE [12]. Dunne et al. (2018) conducted a workshop on test stress and coping strategies, including skills like self-regulatory planning, relaxation, and cognitive reframing. Participants felt more empowered to manage stress during assessments [44]. In light of these insights and the qualitative findings of the present study, it appears that the design and implementation of needs-based interventions for high-stress nursing students prior to examinations can play a pivotal role in alleviating OSCE-related stress.

Nursing students reported significant challenges in managing their time during the OSCE, which contributed to heightened stress levels. The findings of the current study align with previous research indicating that students frequently struggle with the allocation of appropriate time for each station, leading to complaints about insufficient time to complete the necessary procedures [1,5,9,36,45,46]. The literature indicates that time constraints can exacerbate stress levels during high-stakes examinations [1]. According to a descriptive study by Adibone Emebigwine et al. (2022), the perception among students that they lacked sufficient time to complete the OSCE was a primary source of stress. In another qualitative investigation conducted by Nematzad et al. (2023) in Iran, both nursing professors and students reported experiencing time limitations at various stations, which added to their stress [20]. Similarly, Siddique and Ali Shah (2024) identified “time constraints” as a prominent theme in their qualitative study, highlighting that time mismanagement significantly challenges both students and examiners during the OSCE [47]. Due to the pressure of limited time, students often feel rushed, which can lead to confusion regarding the procedural steps they must perform [1]. This rush not only hinders performance but also amplifies stress during the OSCE. These findings underline the importance of effective time management for nursing students during the OSCE. Addressing time-related challenges through targeted training may enhance students’ performance and confidence, ultimately reducing their stress during assessments.

The negative attitudes of nursing students toward examinations, themselves, and their future prospects are significant contributors to the stress they experience during the OSCE. Research supports the notion that students with negative attitudes tend to experience higher stress levels during assessments. Many nursing students develop negative perceptions of the OSCE, often stemming from the intense stress, time constraints, and the pressure of being evaluated on multiple skills at once. Furthermore, concerns regarding the simulated environment’s ability to accurately represent real clinical situations contribute to feelings of inadequacy and the belief that the examination format does not adequately reflect their clinical competence [45]. In this study, nursing students frequently received distressing information about the OSCE from peers who had previously participated, which induced a negative attitude in them and heightened their stress about the OSCE. Additionally, participants expressed significant concern and stress about the fear of failure and its potential consequences. The findings from this study suggest that providing nursing students with accurate and supportive information directly from instructors could alleviate their stress about the OSCE. In addition, in this study, participants emphasized the importance of enhancing self-confidence to combat the challenges posed by the OSCE. Supporting this notion, Ferreira et al. (2020) found that increased self-confidence among physical therapy students helped mitigate the adverse effects of stress on their academic performance during the OSCE [38]. Although high levels of stress can hinder working memory and impede the retrieval of learned information, self-efficacy serves as a protective factor that can counteract these negative impacts [48]. Wong (2008) further proposed that rational beliefs could lead to neutral or positive emotional outcomes [49], a concept leveraged by various researchers to develop cognitive strategies aimed at reducing test stress [42,50,51]. Therefore, providing a supportive environment to address negative attitudes and fears about OSCE exams, present accurate information, and enhance confidence and self-efficacy is essential for reducing nursing students’ OSCE stress.

Based on the findings of this study, establishing a tranquil environment is crucial for alleviating OSCE stress among nursing students. Participants in this study reported heightened stress, particularly in the quarantine or waiting room, a finding supported by the literature [12,28]. In this study, the participants emphasized that creating a calming atmosphere and implementing stress-reduction strategies in this space could significantly mitigate stress before the examination. In this line, several measures were recommended for the waiting area, including informal seating arrangements, the display of positive affirmation posters, and the continuous playback of relaxing music. Such targeted interventions during the pre-examination phase can positively influence students’ emotional states, potentially enhancing performance in high-stakes assessments like the OSCE [44]. Moreover, in this study, the calming presence of instructors (as OSCE supervisors) played a significant role in reducing stress and enhancing performance during the OSCE. In a qualitative study, 65% of participants indicated that the demeanor of examiners could either bolster or undermine their confidence, demonstrating the importance of examiner behavior in influencing student performance [1]. Previous research also highlights that unfriendly examiners contribute to increased stress levels, hindering students’ ability to demonstrate their skills [1,45,52]. Therefore, it is essential for trainers to receive adequate training to foster a supportive atmosphere that promotes student success in clinical evaluations [53]. These findings highlight the critical role of environmental and interpersonal factors in managing test stress among nursing students. Therefore, modifying the waiting environment and promoting supportive examiner behavior may positively affect OSCE stress in nursing students.

### Improving OSCE education and evaluation

Another significant theme identified in this study regarding OSCE-related stress among nursing students was “improving OSCE education and evaluation,” which encompasses sub-themes such as enhancing the educational process, advancing evaluation procedures, and upgrading technological infrastructure. Participants emphasized the need for improvements in both educational and evaluative processes, as well as the provision of related infrastructure to effectively support the examination.

Aligned with the findings of this study, previous research has also highlighted the necessity of enhancing the educational processes of the OSCE [54,55]. Kohansal et al. (2020) noted that the ineffectiveness of clinical education and its misalignment with OSCE requirements pose significant barriers to success in these assessments [54]. Similarly, Nouhi et al. (2021) advocated for the creation of a conducive environment that fosters active and collaborative teaching and learning methods [55]. Qualified nursing education programs have a direct effect on self-efficacy [56]. Higher self-efficacy is associated with lower stress and uncertainty and better performance [57]. In this study, it appears that educational challenges may be associated with feelings of incompetence and reduced self-efficacy among students, which can contribute to the development of OSCE-related stress. These findings underscore the critical need for improvements in the educational framework surrounding the OSCE to enhance student preparedness and alleviate stress.

The findings of this study align with previous research that underscores the pressing need to improve the evaluative processes associated with OSCE [1,20,58]. Based on several studies, participants expressed concerns regarding the limitations of OSCE in effectively assessing students’ competencies [1,20,58]. However, some studies showed that students viewed the OSCE as an effective tool for assessing clinical skills and identifying areas for improvement [59,60]. These contrasting perspectives may stem from variations in participants’ educational backgrounds, differences in evaluative processes, and the resulting diverse experiences encountered by students. In the study by Nematzad et al. (2023), participants suggested implementing varied assessment methods, enhancing the relevance of exam scores for graduation, and adopting approaches to reduce student stress. They also noted that ensuring a strong correlation between OSCE questions and the topics covered in coursework and clinical internships could significantly alleviate stress levels [20], similar to the findings of Zamanzadeh et al. (2021) [61]. The irreversible nature of OSCEs, wherein students have a single opportunity to perform at each station, further contributes to their stress [12]. Additionally, students often report heightened stress at the onset of the OSCE due to unfamiliarity with the examination structure [12,62]. Therefore, to improve the OSCE experience, it is recommended to implement more varied assessment methods and provide preparatory sessions to reduce stress and familiarize students with the exam structure. Additionally, aligning OSCE questions with coursework and clinical internships can help alleviate student stress.

A significant factor contributing to OSCE exam stress among nursing students identified in this study is the inadequacy of educational and technological infrastructure. This aligns with findings from Zamanzadeh et al. (2021), where participants reported shortages in human resources, facilities, equipment, and physical space, highlighting substantial deficiencies in the executive and technical infrastructure supporting OSCE examinations [61]. The study by John (2020) reported that some students faced difficulties communicating with mannequins and models, often perceiving them as lacking realism [46]. These infrastructural limitations lead to perceptions of being ill-prepared, thus exacerbating stress levels among students [61]. To address these challenges, integrating innovative technologies in higher education is essential. Methods like online simulations and blended learning can create a student-centered environment, enhancing both collaborative and individual learning experiences [63,64]. Video simulation, in particular, provides an effective e-learning approach, allowing students to practice clinical skills interactively and familiarize themselves with OSCE scenarios, fostering a positive attitude toward clinical assessments [65]. However, challenges remain regarding the use of simulation tools in nursing education, including issues such as budget, availability of expert staff, and technical support, which participants of this study referred to and need to be addressed. In conclusion, addressing these infrastructural deficiencies is crucial for enhancing the effectiveness of OSCE assessments. By investing in adequate resources and technology, nursing education can better prepare students for clinical evaluations, ultimately reducing stress and improving overall performance.

### Preparing students for the OSCE

Participants in this study recognized “preparing students for the OSCE” as one of the most important themes and challenges related to OSCE exam stress. This central theme includes three sub-themes: “awareness of the exam process,” “previous exam experience,” and “pre-OSCE practice and preparation.” The participants highlighted that reducing OSCE stress among nursing students requires a clear understanding of the exam process, participation in practice sessions, and engagement in OSCE-like simulations.

The findings from this study underscore the importance of raising awareness among nursing students regarding the OSCE exam process as a means to mitigate stress. Other studies align with the findings of this study regarding the positive and effective role of pretest introductory information about the OSCE in reducing exam stress [44,66]. For instance, Fidment (2012) emphasized the importance of providing clear and explicit information about exam expectations and conditions to alleviate students’ apprehensions [66]. Moreover, Dunne et al. (2018) found that conducting an OSCE familiarization workshop significantly reduced test-related stress among nursing students [44]. The OSCE is a unique assessment method that can induce higher levels of stress compared to traditional examination formats [67,68]. This increased stress often stems from students’ unfamiliarity with the OSCE format, especially in summative assessment contexts [69]. Sánchez-Conde and Clemente-Suárez (2021) support this notion, indicating that anticipatory stress is common among students due to the distinctive nature of the OSCE [28]. Literature highlights several ongoing challenges related to this unfamiliar assessment format, including student confusion and stress [53]. To address these challenges, Mojarrab et al. (2020) demonstrated the effectiveness of using slideshows and video presentations to familiarize students with the OSCE process prior to the examination [12]. This approach can help mitigate stress by providing clearer expectations and reducing unfamiliarity with the assessment format. Therefore, it seems that to mitigate OSCE-related stress, it is essential to enhance nursing students’ understanding of the exam format through effective familiarization strategies. Implementing pretest workshops and providing clear information can significantly reduce stress levels and improve student confidence. This proactive approach fosters a supportive learning environment, ultimately enhancing performance during the OSCE.

Participants in this study emphasized the significance of engaging in simulations that closely resemble the OSCE as a means to alleviate exam-related stress. The findings of this study align with the results of other studies [44,70–72]. For example, Young et al. (2014) noted that pre-test OSCE simulations significantly reduced stress levels among students [72]. Furthermore, in the study of Dunne et al. (2018), a ‘mock OSCE’ was designed to replicate the exam environment, demonstrating a positive impact on students’ ability to manage stress [44]. Similarly, Coe and Bryant (2022) found that student-led online mock OSCEs effectively reduce stress among nursing students, contributing to increased confidence and improved retention of clinical skills [70]. Moreover, the results of the Abbasi et al. (2023) study suggest that video simulations can effectively decrease OSCE-related stress among nursing and midwifery students [73]. These findings highlight the importance of familiarization techniques, such as simulations and mock exams, in helping students navigate the stress associated with OSCE assessments.

In the present study, the importance of engaging in practice sessions to prepare nursing students for the OSCE procedures was highlighted as crucial for enhancing performance and reducing stress. Beneficial strategies for OSCE preparation were identified, including various approaches such as repeated practice sessions, lecturer-led theory and practice orientation, and individual or group lab practice [1]. Extensive pre-OSCE practice and preparation are strongly linked to significantly lower stress levels during the actual OSCE exam, as familiarity with the format, procedures, and potential questions helps students feel more confident and less stressed about the unknown aspects of the assessment. By engaging in structured practice sessions, nursing students can become more familiar with the examination format and expectations. This familiarity can alleviate stress and promote a sense of readiness, enabling students to approach the OSCE with greater confidence [1]. Ultimately, such preparation not only improves performance but also fosters a supportive learning environment, equipping students with the skills and mindset necessary for success during the OSCE.

In this study, participants reported high stress levels during the OSCE and emphasized the need for OSCE stress management approaches, including tailored strategies, time management training, addressing negative attitudes, and fostering a peaceful atmosphere. Other key challenges to managing OSCE stress included improving the OSCE’s educational and evaluation methods and upgrading infrastructure. Additionally, better preparation—through raising awareness about the exam, practicing in simulated conditions, and familiarizing students with OSCE procedures—was identified as essential for reducing stress. These findings support Zeidner and Matthews’ (2005) theory, which suggests that test stress is multifaceted, influenced by metacognition, coping strategies, motivation, self-belief, perceived competence, and related beliefs [74]. Addressing these factors comprehensively can lead to more effective interventions for reducing OSCE-related stress, ultimately enhancing students’ performance and well-being.

The findings of this study align with Lazarus’ Stress Theory, which posits that stress emerges from individuals’ cognitive appraisal of potential threats [27]. Nursing students perceive OSCE-related stress as a significant threat, with challenges linked to stress management skills, preparation, and the educational and evaluation processes identified as key stress-inducing factors. These results underscore the need for targeted interventions by nursing school administrators and policymakers to address these challenges. Enhancing students’ coping mechanisms and improving their preparation are critical steps toward mitigating OSCE-related stress effectively.

This qualitative study has several limitations characteristic of qualitative research methodologies. The limited sample size may restrict the depth of insights and fail to encompass the diverse experiences of nursing students and educators. Furthermore, the context-specific nature of the findings, derived from a single university setting, limits the generalizability of the results to broader educational contexts. These limitations highlight the necessity for further research to investigate additional factors influencing OSCE-related stress across various settings.

## Conclusion

This study explored the challenges and needs of nursing students in relation to OSCE exam stress, identifying three main themes: “implementing OSCE stress management approaches”, “improving OSCE education and evaluation”, and “preparing students for the OSCE.” The findings indicate that nursing students experience high levels of OSCE stress, necessitating various management strategies. Furthermore, significant challenges related to OSCE stress include educational and evaluation processes, as well as preparation for the OSCE itself. It appears that addressing these challenges through enhancing self-confidence and competence may effectively alleviate OSCE-related stress. Therefore, nursing education policymakers and faculty authorities should adopt a multidimensional approach to address OSCE-related stress, ultimately improving the psychological well-being and academic performance of nursing students.

## Supporting information

S1 File Semi-structured interview guide used in the study. (PDF)
