# Supplementary material for: Exploring the challenges and needs of nursing students in relation to OSCE exam stress: A qualitative study
Source: PLoS One. 2025 Jul 14;20(7):e0327898. doi: 10.1371/journal.pone.0327898 (PMC12258589; doi:10.1371/journal.pone.0327898)
Supplement: S1 File — (PDF) [file pone.0327898.s001.pdf]

Supporting information

S1 File. Semi-structured interview guide used in the study.

Title: Exploring the challenges and needs of nursing students in relation to OSCE exam stress: a qualitative study.

Study question: What are the challenges and needs of nursing students in relation to Objective Structured Clinical Examination (OSCE) exam stress?

1. Introduction

- The interviewer will introduce themselves and explain the purpose of the interview, including the estimated duration.
- Participants will be assured that all information collected during the interview will be kept strictly confidential, and their identity will remain anonymous.
- Participants will be informed that any details or statements they provide will only be used for the purpose of the research.
- Informed consent will be obtained from the participants, including consent for audio recording.

2. Interview guide

The interview guide is designed to explore the experiences and perspectives of nursing students and instructors regarding OSCE-related stress. The questions aim to identify the main stressors and potential strategies for alleviating stress during the OSCE.

1. **Can you describe any stressful challenges you have faced as a nursing student during the OSCE?**
2. **Which components of the OSCE do you find most stress-inducing, and what do you believe contributes to this stress?**
3. **What forms of support do nursing students require to alleviate their stress related to the OSCE?**
4. **What are your thoughts on the stress experienced by medical students during the OSCE?**
5. **What factors do you think contribute to students' stress during the OSCE?**
6. **How do you feel about being evaluated through the OSCE? Why? (Student)**
7. **What measures could be effective in reducing or eliminating OSCE-related stress for students?**
8. **What topics and strategies do you expect to be presented regarding stress management for the OSCE? (Student)**
9. **Do you have any experience with students under your supervision experiencing OSCE-related stress? If yes, can you elaborate? (Instructor)**

10. **What programs or strategies do you think could help manage this type of stress in students?**
11. **In your opinion, what format would be most suitable for presenting stress management content to students?**

Exploratory follow-up questions will be asked as needed to clarify responses and gain deeper insights, including prompts such as "Can you explain more?", "Can you give an example?", or "What do you mean?"

3. Final remarks

- **Is there anything else you would like to share regarding your experiences and needs in managing OSCE-related stress?**
- **Thank you for your participation and valuable insights.**

Notes:

Follow-up questions may be used to further explore topics based on participant responses.

- The interview will be conducted in a flexible manner, allowing for in-depth exploration of experiences and
